# Entrepreneurial training in public health postgraduate programs: a systematic review of educational approaches

**DOI:** 10.3389/fpubh.2026.1747628

**Published:** 2026-06-23

**Authors:** Nazik M. Nurelhuda, Fadumo Noor, Moetaz El Sergany, Zufishan Alam, Zafar Imam Khan, Sanjai Parahoo

**Affiliations:** 1 School of Health Sciences, Hamdan Bin Mohammed Smart University, Dubai, United Arab Emirates; 2 School of Sustainability and Green Economy, Hamdan Bin Mohammed Smart University, Dubai, United Arab Emirates; 3 Library, Hamdan Bin Mohammed Smart University, Dubai, United Arab Emirates; 4 School of Business and Quality Management, Hamdan Bin Mohammed Smart University, Dubai, United Arab Emirates

**Keywords:** curriculum, education, entrepreneurship, public health, systematic review

## Abstract

The public health sector is undergoing sustained change, shaped by complex health challenges, constrained resources, and evolving employment conditions that may affect the availability of stable and fulfilling career pathways for public health professionals. Within this context, the integration of entrepreneurial competencies into public health training has been proposed as one potential strategy to support innovation, workforce adaptability, and career diversification. This systematic review examined the reported landscape of entrepreneurial training within postgraduate public health programs and described the educational approaches through which such training is delivered. A comprehensive literature search was conducted across eight databases (PubMed, Scopus, ProQuest Central, Cochrane, EBSCO Medline Ultimate, Google Scholar, PsycINFO, and Semantic Scholar) for studies published between 2000 and May 2025. A complementary targeted scan of publicly available Master of Public Health (MPH) and related programs (13–15 August 2025) identified universities explicitly referencing entrepreneurship-related training. Three peer‑reviewed studies met the inclusion criteria, indicating a small and heterogeneous body of empirical work on public health entrepreneurship (PHE) education at the postgraduate level. Across included studies, PHE was inconsistently defined and most often positioned as an elective rather than a core curriculum component. Educational approaches primarily used experiential, team‑based, and mentor‑supported formats and addressed a broad, but not consistently specified, set of competencies, including innovation and opportunity recognition, networking, communication and stakeholder engagement, management and leadership, and basic legal, marketing, and financial knowledge. Outcomes were largely limited to short‑term, self‑reported perceptions, with little evidence on long‑term skill acquisition, career pathways, or workforce‑level effects. Reported constraints included limited faculty expertise and mentorship capacity, restricted institutional resources, and uneven access to entrepreneurship‑supportive ecosystems. Current evidence suggests that postgraduate PHE training is emerging but remains sparsely documented and weakly evaluated. Further research is needed to develop shared competency frameworks, apply more rigorous and longitudinal evaluation designs, and investigate scalable delivery models, including digital and AI‑enabled approaches, with particular attention to equity in access to entrepreneurship training within public health education.

## 1 Introduction

Public Health Entrepreneurship (PHE) has emerged in response to the growing aspiration among public health learners and professionals to adopt more action-oriented approaches to addressing population health challenges (1). PHE can be understood as the application of entrepreneurial methods such as opportunity recognition, rapid experimentation, innovative financing, and partnership building to design, implement, and scale solutions that improve population health and equity. What makes it different from commercial digital health start ups or one off projects is its explicit focus on public value, long term sustainability, and system level impact (2–4).

PHE also shares conceptual boundaries with several related constructs, including public health innovation, social entrepreneurship, intrapreneurship, and commercial health entrepreneurship (3, 5, 6). While these concepts provide important reference points, the extent to which postgraduate public health programs adopt a shared understanding of entrepreneurship remains unclear.

Globally, public health learners have reported increasing fatigue and frustration as they confront rising health challenges without observing timely or sustainable solutions in practice (2). Concurrently, evolving employment landscapes have made it more difficult for public health professionals to identify stable and fulfilling career paths, underscoring the need for adaptive and entrepreneurial approaches to workforce development (7). These challenges are particularly acute for public health professionals affected by conflict, displacement, and humanitarian crises, who often experience abrupt career disruption, loss of professional networks, and restricted access to formal employment in host settings. This confluence of workforce instability and constrained career pathways has, in turn, generated growing interest among public health professionals and trainees in developing entrepreneurial skills to support innovation and adaptability in healthcare settings (1, 8).

In parallel, public health systems continue to face persistent resource constraints, reinforcing the importance of equipping future professionals with entrepreneurial competencies to design cost-effective and sustainable interventions under conditions of limited funding and capacity (9–11).

While entrepreneurship education has been widely examined across disciplines, its application within public health education remains under-explored (12, 13). Despite the established evidence base for entrepreneurship education, there is a pressing need to examine how these pedagogical strategies are translated into public health training contexts (14). Addressing this gap is essential to strengthen understanding of how public health postgraduate programs can effectively prepare trainees for innovative and entrepreneurial roles, ultimately contributing to improved public health outcomes.

Against this backdrop, this systematic literature review aimed to explore the current landscape of entrepreneurial training in public health postgraduate programs, and to describe the approaches through which entrepreneurial training is delivered.

## 2 Materials and methods

This systematic review was conducted and is being reported according to Preferred Reporting Items for Systematic Reviews and Meta-Analyses (PRISMA) guidelines (15). (Checklist provided in Supplementary Table 1). The protocol was registered with the International Prospective Register of Systematic Reviews (PROSPERO; registration ID: CRD42024588688).

### 2.1 Search strategy

The search strategy encompassed a comprehensive review of the literature published from the year 2000 onwards. Reviewing studies published from 2000 onwards is justified as this period captures the integration of entrepreneurship into public health education, driven by technological advancements, global health initiatives, and educational reforms (16).

The search was conducted between 2 and 15 May 2025, without language restrictions, using combinations of keywords and wildcards where applicable. However, only studies published in English were included at the screening stage. The following search concepts were applied: (1) Public Health AND (Education OR Entrepreneurship); and (2) Public Health AND Education AND Entrepreneurship. Details of the full search strategy are provided in Supplementary Table 2.

The search was conducted across the following eight databases to ensure a broad coverage of the relevant literature: PubMed, Scopus, ProQuest Central, Cochrane, EBSCO Medline Ultimate, Google Scholar PsycINFO, and Semantic Scholar. Dissertations, indexed into the databases that met the inclusion criteria, were also searched to retrieve the scholarly articles. The screening was performed using Rayyan (17), a web-based tool for managing systematic reviews.

### 2.2 Inclusion criteria

This review included all studies, reports, dissertations, conference proceedings or grey literature, with no restrictions on design or geographic region, that reported on the development, implementation, or evaluation of entrepreneurial skills in public health postgraduate programs, particularly educational strategies, curricula, or interventions focusing on entrepreneurship in public health. Only publications available in English were included.

### 2.3 Selection process

**Figure 1:**
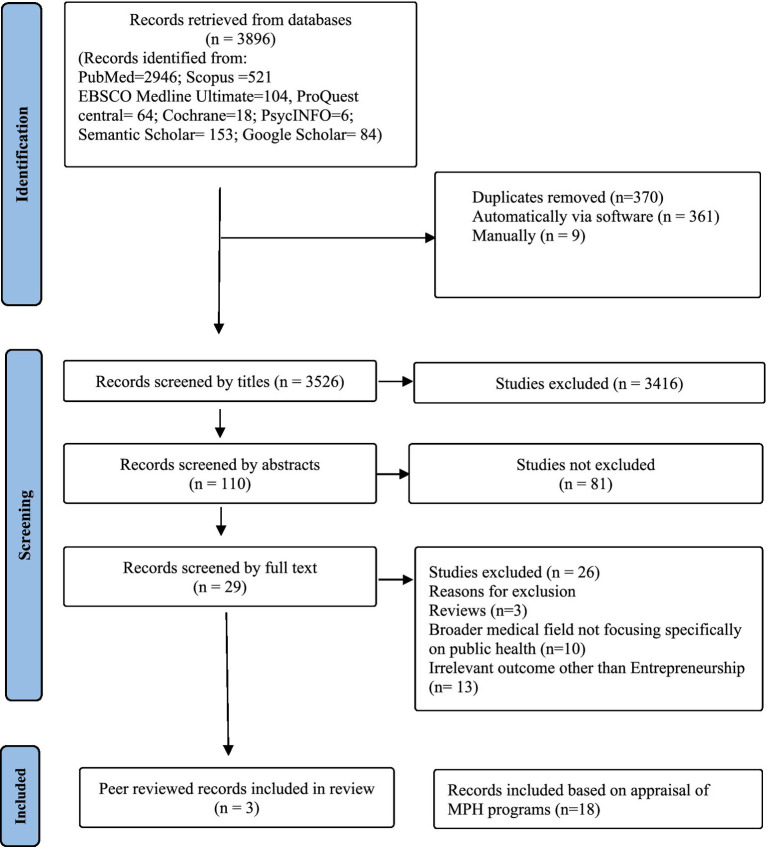
PRISMA flow chart for process of selecting the studies included in the systematic review.

The process involved rigorous, multistage record screening by at least two independent reviewers (NN, FN, MS, and ZA). The retrieved records were imported into the review management software, Rayyan, which facilitated a systematic and blinded screening process. It was followed by record screening at three different levels; title followed by abstract and then the full text. At each level, the records were carefully assessed and those befitting the eligibility criteria and relevance were included. Any disagreement between the reviewers was resolved by a third reviewer. (Figure 1).

### 2.4 Data extraction

For each included article, two reviewers (FN and MS) independently extracted standardized information into a structured Excel form capturing: bibliographic and context variables (Title, Author(s), Year of publication, Country/Region, language, Study design); purpose and content variables (Objective/Aim, sample size, population description, Intervention description, and any stated Framework/Syllabus used to guide curriculum design); delivery variables (Implementation mechanism detailing teaching and learning methods, assessment approaches, delivery timeline, and implementers/instructors); competency variables (Entrepreneurial skills addressed such as opportunity recognition, design thinking, business modelling, leadership/stakeholder engagement, policy navigation, and scaling/impact); results variables (Outcomes/Impact and Learner feedback); and implementation variables (Challenges & barriers, Facilitators, and Author recommendations/guidelines). Where available, notes recorded whether entrepreneurship was embedded as core content, offered as an elective, or linked to a capstone/practicum. Disagreements between reviewers were reconciled by a third reviewer.

### 2.5 Scan of publicly available master of public health programs

To augment the peer-reviewed synthesis, a targeted scan of publicly available descriptions of Master of Public Health (MPH) and closely related postgraduate public-health programs was conducted by lead author (NN) between 13 and 15 August 2025, with assistance from ChatGPT (OpenAI, San Francisco, CA, United States) to refine search terms and keyword combinations. All program entries and extracted fields were identified, verified, and recorded manually by the reviewer against the most recent information available on institutional websites and official program materials. The aim was to identify examples of entrepreneurship training visible in university materials available online. University websites and online catalogs using structured keyword strings (e.g., “MPH entrepreneurship,” “public health innovation course”, “social entrepreneurship public health”, “capstone entrepreneurship public health”), site-restricted queries (e.g., site:.edu, site:.ac.uk), and within-site search tools to locate program pages, handbooks/syllabi, course catalogs, innovation hub pages, and cross-registration policies. This program scan was not intended as an evidence stream equivalent to the systematic review, but as a contextual supplement illustrating how entrepreneurship training is represented in postgraduate public health programs. Its findings are presented descriptively to support interpretation of the review themes, rather than as empirical evidence.

Inclusion criteria: Postgraduate public-health degrees (MPH or clearly allied master’s) that (a) list entrepreneurship or innovation content as core/required, concentration/track, elective, or capstone/practicum; or (b) provide documented, for-credit access to entrepreneurship coursework via cross-registration. We also recorded adjacent opportunities (co-curricular incubators/accelerators or related non-MPH degrees) when they were explicitly positioned for MPH students; these were flagged as adjacent rather than embedded. Exclusion criteria: Programs without any documented entrepreneurship/innovation component; undergraduate offerings; and non-health entrepreneurship degrees not positioned for MPH students. For each included program we extracted: institution, country/region, degree/track, delivery format (on-campus/online/blended), entrepreneurship offering type (core/concentration/elective/capstone), embedded vs. adjacent classification, evidence of an entrepreneurship-related capstone, full URL(s), and brief notes. All entries were verified against the most recent public catalog or page available at the time of the scan, with ambiguous cases labeled, unclear. This program scan was purposive rather than exhaustive and was intended to provide contextual illustration alongside the findings of the systematic literature review. As it relied on publicly available website information, it is subject to limitations including English-language bias, variability in the level of curricular detail reported online, and the potential for rapid program changes over time.

### 2.6 Data synthesis plan

The primary synthesis was based on the peer-reviewed studies included in the systematic review. Findings from the program scan were used as contextual illustrations to support interpretation of the themes identified in the literature. The analysis followed a thematic synthesis approach. Initial coding was conducted inductively on the three included studies to identify recurring concepts related to the definition, integration, delivery, and outcomes of public health entrepreneurship training. These codes were iteratively grouped into higher-order themes through team discussion and refinement. A thematic framework was then developed, comprising six domains: conceptual definitions, curriculum integration, educational approaches, entrepreneurial skills, outcomes, and challenges/facilitators. Each included study was systematically mapped to these domains, and findings were narratively synthesized across themes. Given the limited number and heterogeneity of included studies, the synthesis focused on identifying patterns, areas of convergence and divergence, and gaps in the evidence, rather than quantifying effects or drawing causal inferences.

## 3 Results

Overall, 3,896 records were retrieved, including 521 articles from Scopus, 104 from EBSCO Medline Ultimate, 2,946 from PUBMED, 64 from ProQuest Central, 18 from Cochrane, 6 from PsycINFO, 153 from Semantic Scholar, and 84 from Google Scholar. From the included records 370 duplicates were removed either automatically by the software (*n* = 361) or manually (*n* = 9). After screening titles of the remaining 3,526 records, 110 records were included for abstract screening against the eligibility criteria. Of the reviewed records, 29 were found to be eligible and relevant for full text screening. After conducting full text screening, three records were finally included in the systematic review (Figure 1: PRISMA Diagram, Table 1).

**Table 1:** Summary of the three included studies, outlining the study country, design, population, key findings and study limitations.

Title	Authors (year)	Country/context	Study design	Population/setting	Aim/focus	Key findings/relevance	Study limitations
Public health entrepreneurs: training the next generation of public health innovators	Hernández et al. (19)	United States (Columbia University)	Commentary/Perspective	Public health graduates and workforce	To advocate for entrepreneurship training in public health	Defines public health entrepreneurship; identifies funding and employment challenges; proposes integrating entrepreneurship into public health training	Not empirical; perspective piece
Curricula and resources related to social entrepreneurship and public health innovation within schools of public health in the United States	Hyde et al. (18)	United States (survey of 15 SPH)	Cross-sectional survey	Faculty in schools of public health	To assess social entrepreneurship and innovation offerings in public health curricula	Few structured programs exist; barriers include funding and faculty capacity; identified opportunities for expansion	Small sample size (15 schools), US only
Public health entrepreneurship: a novel path for training future public health professionals	Becker et al. (2)	United States (UT school of public health; harvard SPH)	Qualitative (focus groups)	Graduate public health students (*n* = 29)	To explore perceptions of public health entrepreneurship training	Students saw entrepreneurship as skills-driven, action-oriented, and aligned with CEPH competencies	Pilot programs only, small sample

Backward citation searching of the three records did not identify any additional eligible articles, reinforcing the limited empirical evidence base for entrepreneurial training within public health education.

### 3.1 Assessment of risk of bias and study quality

The qualitative study by Becker et al. (2) was appraised using the Critical Appraisal Skills Programme (CASP) Qualitative Checklist and demonstrated moderate methodological quality, with clearly stated aims and appropriate qualitative methods; however, reliance on self-reported data, a small and self-selected sample, potential selection bias, and limited reporting on researcher reflexivity increased the risk of bias. The cross-sectional institutional mapping study by Hyde et al. (18) was assessed using the Joanna Briggs Institute (JBI) Checklist for Analytical Cross-Sectional Studies and was judged to have a moderate to high risk of bias, primarily due to a low response rate, non-random sampling, and reliance on respondent-reported information without independent curricular verification. The commentary by Hernández et al. (19) was appraised using the JBI Checklist for Text and Opinion Papers; while it offers valuable conceptual insights and recommendations, the absence of an empirical design, systematic data collection, or outcome evaluation confers a high risk of bias with respect to effectiveness or impact claims. Across the included studies, common methodological limitations included the absence of standardized outcome measures, limited assessment of educational effectiveness, and a lack of longitudinal follow-up. No studies were excluded on the basis of quality alone; however, these limitations were taken into account when interpreting the findings.

**Figure 2:**
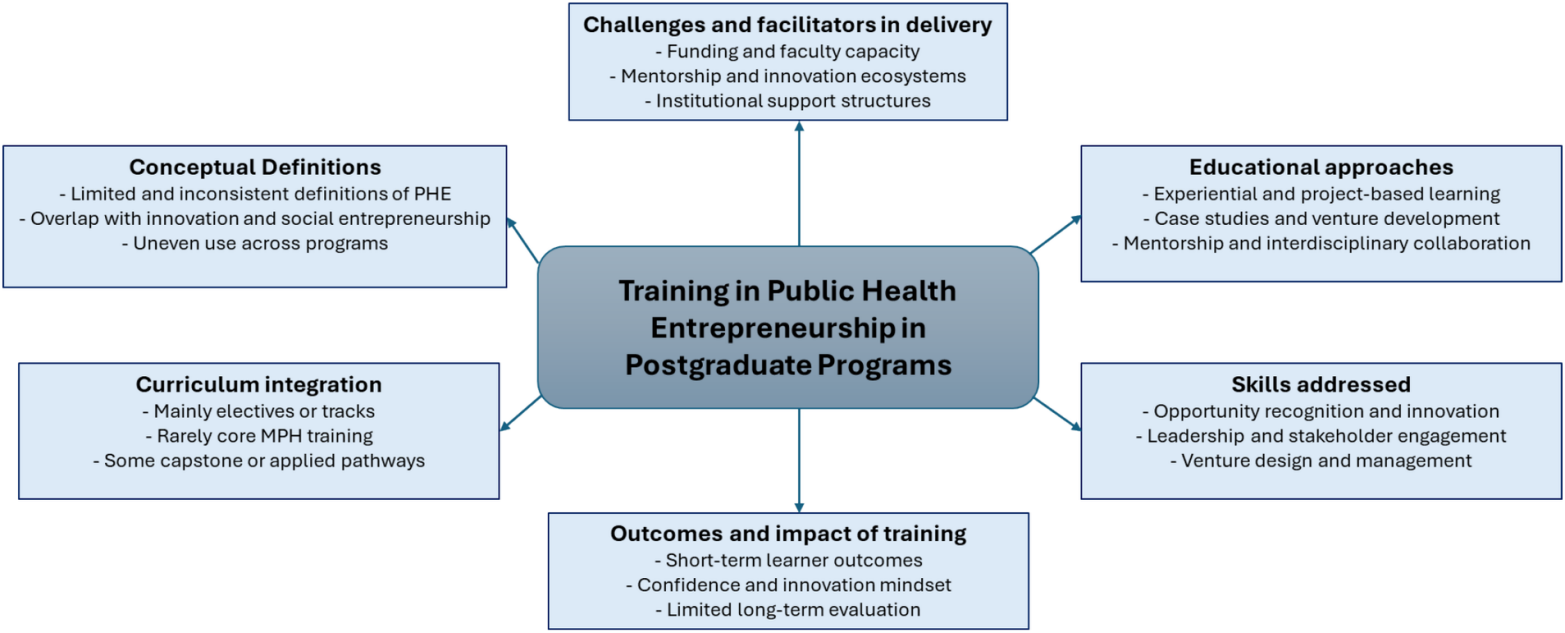
Visual synthesis of thematic domains identified in the review.

The scan yielded 18 global MPH and related postgraduate public health programs that offer some form of PHE training (Table 2). In the following section, we present the analysis of these resources focusing on six themes. Figure 2 provides a visual synthesis of the thematic domains identified in the review. Detailed analysis is presented in Supplementary Table 3.

**Table 2:** Summary of results from the program scan, presenting institution and program name, country or region, type of integration, entrepreneurship focus or skills addressed, and educational approach.

Institution/program	Country/region	Integration type	Entrepreneurship focus/skills	Pedagogical approach
Arizona State University–MPH in Public Health Technology	United States/North America	Core (Embedded)	Design thinking, business model creation, health-tech innovation	Applied, project-based learning
Columbia University Mailman School of Public Health	United States/North America	Embedded	Social venture development, pitching, innovation strategy	Coaching, workshops, Fast-Pitch competition
American University of Beirut–MPH (Social Entrepreneurship elective)	Lebanon/MENA	Elective	Social enterprise creation, community-based innovation	Mentored studio seminar
Harvard T. H. Chan School of Public Health + Harvard Business School	United States/North America	Elective/Cross-registration	Business model design, innovation strategy, scaling solutions	Case method, field projects
Johns Hopkins University–Bloomberg School of Public Health	United States/North America	Cross-track integration	Social innovation and design thinking within selected MPH pathways	Project-based, interdisciplinary
University of Toronto–Dalla Lana School of Public Health	Canada/North America	Cross-registration	Innovation and entrepreneurship electives across university ecosystem	Active, cross-disciplinary learning
University of British Columbia–School of Population and Public Health	Canada/North America	Cross-registration	Access to entrepreneurship ecosystem (Sauder School, UBC HATCH)	Experiential, elective-based
Yale School of Public Health (via Yale School of Management)	United States/North America	Embedded + Elective	Opportunity recognition, leadership for health equity, venture design	Case-based, MBA-style project work
UNC Gillings School of Global Public Health	United States/North America	Embedded	Social/commercial entrepreneurship, stakeholder engagement	Case studies, applied projects
CUNY Graduate School of Public Health and Health Policy	United States/North America	Embedded	Problem discovery, prototyping, market validation	Studio course, accelerator linkages
University of California, Berkeley–Concurrent MBA/MPH	United States/North America	Dual Degree (Elective)	Strategic innovation in health, operations, marketing	Integrated MBA–MPH coursework
George Washington University–Online MPH	United States/North America	Elective	Innovation and entrepreneurship topics in selective modules	Online, interdisciplinary
Northeastern University–MPH	United States/North America	Elective	Design thinking, systems innovation	Interdisciplinary elective courses
University of Southern California–MPH + MS in Social Entrepreneurship	United States/North America	Dual Degree	Social venture design, fundraising, impact measurement	Integrated coursework, applied projects
University of Cape Town–Bertha Center/SIHI Collaboration	South Africa/Sub-Saharan Africa	Co-curricular	Health systems innovation, social entrepreneurship	Practice-based projects, incubator
University of Johannesburg–MPH	South Africa/Sub-Saharan Africa	Potential elective	Innovation management (under development)	Not documented
Karolinska Institutet–Freestanding Courses	Sweden/Europe	Elective	Digital health entrepreneurship, biotech commercialization	Project-based, freestanding courses
University of Auckland–MPH (Innovation Workshops)	New Zealand/Oceania	Co-curricular	Innovation process, ideation, design sprints	Maker-space, workshops, incubator

### 3.2 Conceptual definitions of entrepreneurship in public health postgraduate programs

Across the reviewed literature, explicit definitions of entrepreneurship in public health education were limited. Hernández et al. (19) provide the most direct conceptualization, framing public health entrepreneurship as a career path that achieves a “double bottom line” of economic self-sufficiency and social impact, adapted from Dees’ foundational work in social entrepreneurship. Becker et al. (2) define it as the application of entrepreneurial skills to advance public health missions. In contrast, Hyde et al. (18) did not offer a formal definition but reported substantial variation in how entrepreneurship and social innovation are conceptualized across schools of public health. Program scans reveal similar ambiguity, with only a few institutions such as Arizona State University, American University of Beirut, and Columbia University Mailman School providing definitions in public materials. Others, including Harvard Chan and University of Toronto, reference related skills without labeling them as entrepreneurship.

### 3.3 Curriculum integration

Entrepreneurship is integrated into public health curricula through diverse formats. Becker et al. (2) describe pilot courses at Council on Education of Public Health (CEPH)-accredited U. S. schools using case-based learning and team projects. Hernández et al. (19) introduced the Public Health Innovators Seminar Series at Columbia University. Hyde et al. (18) report uneven distribution of entrepreneurship courses across U. S. schools, often dependent on external funding. Program scans show that Arizona State University embeds entrepreneurship in its MPH curriculum with a dedicated capstone. Johns Hopkins integrates social innovation into designated tracks. Elective and cross-registration options are available at Harvard Chan, University of Toronto, UBC and Karolinska. Applied pathways include Columbia Mailman’s Fast-Pitch, NUS TechLaunch’s venture-building model, and the University of Cape Town’s practice-based innovation projects.

### 3.4 Educational approaches

Educational approaches favor experiential, team-based, and mentor-supported learning. Becker et al. (2) use case methods, guest experts, and venture formation projects. Hernández et al. (19) use seminar formats to expose students to entrepreneurial pathways. Hyde et al. (18) highlight sustainability challenges due to funding and staffing. Program examples include Columbia Mailman’s Fast-Pitch, ASU’s design-thinking curriculum, AUB’s mentored studio formats, and NUS TechLaunch’s incubator-style training. Karolinska focuses on commercialization and IP development. Harvard Chan uses case methods and field projects. Institutions such as Toronto, UBC, UCL, Copenhagen, Maastricht, LSHTM, Melbourne, Sydney, Queensland, and Auckland offer electives using active pedagogies.

### 3.5 Entrepreneurial skills addressed

The literature identifies a broad set of entrepreneurial skills. Hyde et al. (18) highlight innovation, networking, and mentorship. Hernández et al. (19) identify legal, marketing, and finance domains. Becker et al. (2) note gaps in team formation and organizational management. Communication and stakeholder engagement are emphasized by Becker et al. (2) Evaluation and accountability are included by Hernández et al. (19) and Becker et al. (2).

### 3.6 Outcomes and impact

Evidence on outcomes was limited and primarily perception-based. Becker et al. (2) reported that students viewed entrepreneurship training as aligned with recent CEPH competency reforms and as addressing unmet needs related to implementation, collaboration, and sustainability of public health solutions. However, outcomes were based on student perceptions rather than objective measures of skill acquisition or venture success. Hyde et al. (18) highlighted challenges in sustaining entrepreneurship initiatives, noting that programs often lacked long-term funding and institutionalization. Hernández et al. (19) suggest entrepreneurship could diversify career paths. Program-level outcomes include Columbia Mailman and ASU’s applied training models, Central American Fellowship’s real-world implementation, and Thailand’s validated curriculum.

### 3.7 Challenges and facilitators

Challenges include resource constraints such as time, mentorship, and funding (18). Faculty expertise are limited, contributing to the fragility of entrepreneurship-related initiatives within schools of public health (18). Siloed training environments and weak translation pathways between academic public health education and applied innovation ecosystems further hinder the development of entrepreneurial competencies (2). Facilitators are largely described in aspirational or recommended terms rather than as empirically evaluated enablers. Structured mentorship, multi-sector networks, and continuing peer communities are identified as important supports for entrepreneurship-oriented training (2, 19). Access to seed capital and institutional mechanisms that support double-bottom-line objectives are highlighted as potential facilitators for sustaining entrepreneurial activity within public health education (19). Overall, the included studies emphasize the need for cultural and institutional incentives that recognize entrepreneurship as a complement to traditional public health training, rather than a replacement (2).

## 4 Discussion

Given the predominantly descriptive and perceptual nature of the available evidence, interpretations emphasize patterns, gaps, and variability rather than causal or effectiveness claims.

This qualitative systematic review, complemented by a program scan, explored how the emerging field of PHE is defined, integrated, and taught in postgraduate public health programs. While interest in PHE appears to be growing, its adoption remains uneven.

Our review identified a lack of definitional clarity. This ambiguity likely reflects the interdisciplinary origins of PHE, which draws on traditions from social entrepreneurship, innovation studies, and organizational intrapreneurship. It poses two risks for the academic field (3). Firstly, it may hinder alignment across institutions and secondly it may contribute to inconsistencies in how competencies are interpreted and assessed. Without a shared conceptual framework, PHE risks being treated as a loosely defined set of skills and competencies (20).

PHE was often positioned as a niche track rather than a core component of public health training. Although multiple routes exist to integrate entrepreneurship (21), reflecting a diversity in approaches, it remains optional in most programs. This elective-only status may limit exposure to self-selecting learners, reducing workforce wide exposure. Mandatory models are feasible. Examples from the program scan, such as Arizona State University’s required capstone, and Columbia Mailman’s structured Innovation pathways, illustrate that more integrated models are possible. However, there is currently limited empirical evidence to assess their effectiveness.

Entrepreneurship education in public health is predominantly experiential and mentor-supported, often using design thinking, case studies, hackathons, or incubator-style cohorts. These strategies are consistent with adult learning principles and appear well suited to applied, practice-oriented training (22). However, sustainability remains a challenge, particularly in relation to funding, time, and access to mentorship (23, 24). Sustainability also depends on institutional commitment and regional ecosystems (25). Observations from the program scan suggest that institutions with established innovation hubs (e.g., Karolinska, NUS) may be better positioned to deliver sustained mentorship than resource-constrained schools in LMICs, although this has not been systematically evaluated.

The review highlights a broad range of entrepreneurial skill domains: opportunity recognition, venture creation, leadership and management, communication, and impact evaluation. While some overlap exists across programs, few explicitlyarticulate progression levels (for example, from novice to advanced) or map these skills to standardized competency frameworks (26). Some programs emphasize business and economic literacy (e.g., Harvard Chan/HBS, Karolinska), while others focus on social venture design and stakeholder engagement (e.g., AUB, UCT). This diversity is valuable but may also contribute to fragmentation. Without standardized progression models, learners may graduate with uneven or incomplete entrepreneurial preparation (27). Embedding entrepreneurship into the core MPH curricula, supported by competency-based milestones (28, 29), could provide a more structured approach to skill development. Existing public health workforce competency frameworks already emphasize domains such as systems thinking, leadership, partnership development, and innovation that overlap with competencies associated with entrepreneurial practice. For example, the WHO–ASPHER competency framework highlights capacities related to innovation, strategic leadership, and collaborative problem-solving that may provide an entry point for integrating entrepreneurial competencies within public health education (30). Rather than constituting a separate domain, PHE may therefore be articulated across existing competency areas. Programs like ASU and Columbia can serve as models.

Conceptually, public health entrepreneurship can also be situated in relation to adjacent constructs while maintaining a distinct focus. While social entrepreneurship emphasizes social impact (6) and intrapreneurship focuses on innovation within organizations (5), public health entrepreneurship integrates opportunity recognition, implementation, and scaling within a population health context (3). These capabilities can be aligned with existing public health competency domains. For example, opportunity recognition aligns with systems thinking; implementation with program planning; stakeholder engagement with leadership and partnership; and scaling with policy and strategic leadership. This alignment is consistent with established public health competency frameworks that emphasize integrated domains such as leadership, systems thinking, and governance (30). This alignment further supports the interpretation of entrepreneurship as a cross-cutting application of existing competencies rather than a distinct domain. Further work is needed to more systematically articulate and validate these mappings within competency-based education frameworks. Our review suggests some short-term perceived benefits: students report increased confidence, projects secure seed funding or political support, and programs validate curricular models through psychometric methods (31, 32). However, outcome measurement remains largely based on proximal indicators such as satisfaction surveys and self-reported confidence (33). Long-term evidence of career trajectories, venture development, or public health outcomes is limited. Few programs report start-ups formed, funding raised, or innovations implemented at scale. Importantly, the absence of longitudinal evaluation should not be interpreted as evidence that entrepreneurship training is ineffective; rather, it reflects the early stage of research in this area and constrains conclusions regarding long-term impact (34, 35). Future research may benefit from developing standardized, longitudinal evaluation approaches that capture both individual competencies and systemic contributions (34).

Common challenges included resource constraints, limited faculty expertise, and reliance on electives (36). These challenges may be more pronounced in LMIC contexts, where institutional capacity is weaker (18, 37). This may contribute to inequities in access to entrepreneurship training, with more resourced institutions better positioned to sustain such initiatives (38). Facilitators identified include innovation hubs, cross-faculty ecosystems, mentorship networks, and small-scale funding opportunities. For example, Harvard and UCL benefit from cross-campus innovation hubs, while Columbia leverages an established pitch ecosystem. LMIC programs such as UCT show that regional networks and community partnerships can also act as enablers. However, the transferability of these models remain underexplored (39): few studies evaluate how entrepreneurship education can be adapted to low-resource settings.

These observations also point to potential areas for future development. Online delivery models may offer opportunities to expand access to entrepreneurial training by reaching learners in underserved or resource-constrained contexts without the heavy infrastructure demands of in-person incubators. Emerging AI-enabled platforms may also support personalized learning (e.g., adaptive modules on business modeling, regulatory navigation, or design thinking) and mentorship (40, 41). AI-driven analytics could also strengthen the currently weak outcome measurement landscape by tracking learner progression and simulating venture scenarios, thereby providing competency-based evidence of skill acquisition. However, evidence on the effectiveness of such approaches within public health education remains limited and warrants further investigation.

### 4.1 Methodological appraisal

This review followed PRISMA guidelines (15), registered a protocol with PROSPERO, and employed a comprehensive, multi-database search spanning both peer-reviewed and grey literature. Rigorous multistage screening with independent reviewers enhanced reliability, while the inclusion of dissertations and conference proceedings broadened the evidence base. The targeted program scan provided contextual illustration of current practice, supporting interpretation of the findings. Nonetheless, several limitations should be noted. Despite broad searching, the evidence base remains small (three records), limiting generalizability. The program scan was purposeful rather than exhaustive, subject to English-language and website-reporting bias, and may not reflect rapidly changing curricula. In addition, the systematic review was limited to studies published in English, which may have resulted in the exclusion of relevant studies published in other languages. Reliance on self-reported outcomes in the included studies also constrains confidence in reported impact. Together, these limitations highlight the need for more sound primary research and transparent reporting of entrepreneurship training in public health education.

This review suggests that entrepreneurial training in postgraduate public health education is an emerging but still evolving area. Definitions remain inconsistent, integration is often elective, and pedagogical approaches, while innovative, face sustainability challenges. While early indications point to potential value in supporting skills related to innovation and implementation, the current evidence base remains limited and largely descriptive. Further empirical research is needed to better understand the role, effectiveness, and optimal integration of entrepreneurship within public health education.

### 4.2 Implications for policy and practice

The findings highlight growing interest in PHE as a potential area of competency development within the public health workforce. Policy makers, academic leaders, and funders may consider exploring how entrepreneurial competencies could be incorporated within existing public health education and workforce development frameworks. Accrediting bodies may also play a role in examining how such competencies could be incorporated. Universities may explore approaches that extend beyond elective offerings toward more structured opportunities. Investment in mentorship networks, innovation hubs, and cross-disciplinary collaboration may help support the development of entrepreneurial capacity.

## Data Availability

Publicly available datasets were analyzed in this study. This data can be found at: https://hbmsu-my.sharepoint.com/:x:/g/personal/nsuleiman_hbmsu_ac_ae/ESByGCPpij1FkqBj_R4kQt4BiSAwhMrFkKdozWWAh7jubg?e=hxfMBE.
