# Supplementary material for: Entrepreneurial training in public health postgraduate programs: a systematic review of educational approaches
Source: Front Public Health. 2026 Jun 23;14:1747628. doi: 10.3389/fpubh.2026.1747628 (PMC13337808; doi:10.3389/fpubh.2026.1747628)
Supplement: Supplementary file 1 [file Table_1.docx]

Supplementary Table 1: PRISMA Checklist for the systematic review

| Supplementary Table 3: PRISMA ChecklistSection and Topic | Item # | Checklist item | Location where item is reported |
| --- | --- | --- | --- |
| TITLE | | |  |
| Title | 1 | Identify the report as a systematic review. | Title, Page 1 |
| ABSTRACT | | |  |
| Abstract | 2 | See the PRISMA 2020 for Abstracts checklist. | Abstract, Page 1 |
| INTRODUCTION | | |  |
| Rationale | 3 | Describe the rationale for the review in the context of existing knowledge. | Introduction, Page 2 |
| Objectives | 4 | Provide an explicit statement of the objective(s) or question(s) the review addresses. | Introduction Page 2,3 |
| METHODS | | |  |
| Eligibility criteria | 5 | Specify the inclusion and exclusion criteria for the review and how studies were grouped for the syntheses. | Methods Page 3,4,5 |
| Information sources | 6 | Specify all databases, registers, websites, organisations, reference lists and other sources searched or consulted to identify studies. | Methods Page 3, 4 |
| Search strategy | 7 | Present the full search strategies | Supplementary Table 2 |
| Selection process | 8 | Specify the methods used to decide whether a study met the inclusion criteria of the review | Methods Page 3,4 |
| Data collection process | 9 | Specify the methods used to collect data | Methods Page 4 |
| Data items | 10a | List and define all outcomes | Methods Page 4,5 |
|  | 10b | List and define all other variables | Methods Page 4,5 |
| Study risk of bias assessment | 11 | Specify the methods used to assess risk of bias | Methods Page 5,6 |
| Effect measures | 12 | Specify for each outcome | Not applicable |
| Synthesis methods | 13a | Describe the processes used to decide which studies were eligible for each synthesis | Methods Page 5 |
|  | 13b | Describe any methods required to prepare the data | Not applicable |
|  | 13c | Describe any methods used to tabulate or visually display results of individual studies and syntheses. | Methods Page 5,6 |
|  | 13d | Describe any methods used to synthesize results and provide a rationale for the choice(s). | Not applicable |
|  | 13e | Describe any methods used to explore possible causes of heterogeneity among study results | Not applicable |
|  | 13f | Describe any sensitivity analyses conducted to assess robustness of the synthesized results. | Not applicable |
| Reporting bias assessment | 14 | Describe any methods used to assess risk of bias due to missing results in a synthesis | Not applicable |
| Certainty assessment | 15 | Describe any methods used to assess certainty | Not applicable |
| RESULTS | | |  |
| Study selection | 16a | Describe the results of the search and selection process | Results Page 5,6, Figure 1 |
|  | 16b | Cite studies that might appear to meet the inclusion criteria | Figure 1 |
| Study characteristics | 17 | Cite each included study and present its characteristics. | Table 1, Table 2 |
| Risk of bias in studies | 18 | Present assessments of risk of bias for each included study. | Methods, Page 5,6 |
| Results of individual studies | 19 | For all outcomes, present, for each study: (a) summary statistics for each group (where appropriate) and (b) an effect estimate and its precision (e.g. confidence/credible interval), ideally using structured tables or plots. | Not applicable |
| Results of syntheses | 20a | For each synthesis, briefly summarise the characteristics and risk of bias among contributing studies. | Pages 6, 7,8. Table 1, Table 2  Supplementary Table 3 |
|  | 20b | Present results of all statistical syntheses conducted. If meta-analysis was done, present for each the summary estimate and its precision (e.g. confidence/credible interval) and measures of statistical heterogeneity. If comparing groups, describe the direction of the effect. | Not applicable |
|  | 20c | Present results of all investigations of possible causes of heterogeneity among study results. | Not applicable |
|  | 20d | Present results of all sensitivity analyses conducted to assess the robustness of the synthesized results. | Not applicable |
| Reporting biases | 21 | Present assessments of risk of bias due to missing results (arising from reporting biases) for each synthesis assessed. | Not applicable |
| Certainty of evidence | 22 | Present assessments of certainty (or confidence) in the body of evidence for each outcome assessed. | Not applicable |
| DISCUSSION | | |  |
| Discussion | 23a | Provide a general interpretation of the results in the context of other evidence. | Discussion Page 8,9 |
|  | 23b | Discuss any limitations of the evidence included in the review. | Discussion Page 10 |
|  | 23c | Discuss any limitations of the review processes used. | Discussion Page 10 |
|  | 23d | Discuss implications of the results for practice, policy, and future research. | Discussion Page 11 |
| OTHER INFORMATION | | |  |
| Registration and protocol | 24a | Provide registration information for the review, including register name and registration number, or state that the review was not registered. | Methods Page 3 |
|  | 24b | Indicate where the review protocol can be accessed, or state that a protocol was not prepared. | Not applicable |
|  | 24c | Describe and explain any amendments to information provided at registration or in the protocol. | Not applicable |
| Support | 25 | Describe sources of financial or non-financial support for the review, and the role of the funders or sponsors in the review. | Page 11 |
| Competing interests | 26 | Declare any competing interests of review authors. | Page 11 |
| Availability of data, code and other materials | 27 | Report which of the following are publicly available and where they can be found: template data collection forms; data extracted from included studies; data used for all analyses; analytic code; any other materials used in the review. | Page 11 |
