# Supplementary material for: Entrepreneurial training in public health postgraduate programs: a systematic review of educational approaches
Source: Front Public Health. 2026 Jun 23;14:1747628. doi: 10.3389/fpubh.2026.1747628 (PMC13337808; doi:10.3389/fpubh.2026.1747628)
Supplement: Supplementary file 2 [file Table_2.docx]

Supplementary Table 2: Search strategy for the review

| Search strings (General) | entrepreneurship OR entrepreneur OR startup OR “new business” OR innovator OR inventor OR “small business” OR innovation OR “new service”  AND  education OR program OR university OR course OR diploma OR module OR college  AND  “public health” OR “community medicine” OR “community health” OR “population health” OR healthcare OR “health system” OR “health care” |
| --- | --- |
| Scopus (n=521) | ( TITLE ( entrepreneurship OR entrepreneur OR startup OR {new business} OR innovator OR inventor OR {small business} OR innovation OR {new service} ) AND TITLE-ABS-KEY ( education OR program OR university OR course OR diploma OR module OR college ) AND TITLE ( {public health} OR {community medicine} OR {community health} OR {population health} OR healthcare OR {health system} ) ) |
| EBSCO Medline ultimate (n=104)  Limit: peer reviewed | TI (entrepreneurship OR entrepreneur OR startup OR “new business” OR innovator OR inventor OR “small business” OR innovation OR “new service”) AND TI (education OR program OR university OR course OR diploma OR module OR college) AND TI (“public health” OR “community medicine” OR “community health” OR “population health” OR healthcare OR “health system” OR “health care”) |
| PubMed (n=2946) | ((entrepreneurship[Title/Abstract] OR entrepreneur[Title/Abstract] OR startup[Title/Abstract] OR "new business"[Title/Abstract] OR innovator[Title/Abstract] OR inventor[Title/Abstract] OR "small business"[Title/Abstract] OR innovation[Title/Abstract] OR {new service}[Title/Abstract]) AND (education[Title/Abstract] OR program[Title/Abstract] OR university[Title/Abstract] OR course[Title/Abstract] OR diploma[Title/Abstract] OR module[Title/Abstract] OR college[Title/Abstract])) AND ("public health"[Title/Abstract] OR "community medicine"[Title/Abstract] OR "community health"[Title/Abstract] OR "population health"[Title/Abstract] OR healthcare[Title/Abstract] OR "health system"[Title/Abstract]) |
| ProQuest Central (n=64)  Limit: peer reviewed | title(entrepreneurship OR entrepreneur OR startup OR "new business" OR innovator OR inventor OR "small business" OR innovation OR "new service") AND title(education OR program OR university OR course OR diploma OR module OR college) AND title("public health" OR "community medicine" OR "community health" OR "population health" OR healthcare OR "health system" OR "health care") |
| Cochrane (n=18)  Limit: title, abstract | entrepreneurship OR entrepreneur OR startup OR “new business” OR innovator OR inventor OR “small business” OR innovation OR “new service” in Title Abstract Keyword AND education OR program OR university OR course OR diploma OR module OR college in Title Abstract Keyword AND “public health” OR “community medicine” OR “community health” OR “population health” OR healthcare OR “health system” OR “health care” in Title |
| Psycinfo (n=6) | entrepreneurship OR entrepreneur OR startup OR “new business” OR innovator OR inventor OR “small business” OR innovation OR “new service” AND Keywords: education OR Keywords: program OR Keywords: university OR Keywords: course OR Keywords: diploma OR Keywords: module OR Keywords: college AND Keywords: “public health” OR “community medicine” OR “community health” OR “population health” OR healthcare OR “health system” OR “health care” |
| Semantic Scholar (n= 153) | entrepreneurship OR entrepreneur OR startup OR "new business" OR innovator OR inventor OR "small business" OR innovation OR "new service" AND education OR program OR university OR course OR diploma OR module OR college AND "public health" OR "community medicine" OR "community health" OR "population health" OR healthcare OR "health system" OR "health care" |
| Google Scholar (84) | allintitle: education AND entrepreneurship AND "public health" OR healthcare OR health OR medicine" |

.
