# Supplementary material for: Entrepreneurial training in public health postgraduate programs: a systematic review of educational approaches
Source: Front Public Health. 2026 Jun 23;14:1747628. doi: 10.3389/fpubh.2026.1747628 (PMC13337808; doi:10.3389/fpubh.2026.1747628)
Supplement: Supplementary file 3 [file Table_3.docx]

Supplementary Table 3. Integration of evidence from the systematic review and targeted scan of MPH and postgraduate public health programs: convergences and gaps between research and practice.

| Theme | Evidence from Peer-Reviewed Literature | Program Scan Examples | Insights |
| --- | --- | --- | --- |
| Definition of Entrepreneurship | Hernández et al. (2014) define public health entrepreneurship as achieving a 'double bottom line' of economic self-sufficiency and social impact. Becker et al. (2019) describe it as applying entrepreneurial skills to advance public health. Hyde et al. (2024 discuss it within social innovation. | Arizona State University defines entrepreneurship as designing novel public health solutions. American University of Beirut uses 'social entrepreneurship' for market-based health equity strategies. Columbia Mailman includes entrepreneurship in Fast-Pitch initiatives. Harvard Chan and University of Toronto reference related skills without explicit definitions. | Lack of definitional clarity across literature and practice suggests a conceptual gap that may hinder curriculum alignment and competency measurement. |
| Curriculum Integration | Becker et al. (2019) describe pilot courses with case-based learning. Hernández et al. (2014) introduced a seminar series at Columbia. Hyde et al. (2024) report uneven distribution of entrepreneurship courses. | ASU embeds entrepreneurship in MPH curriculum with a capstone. Johns Hopkins integrates social innovation into tracks. Electives and cross-registration at Harvard Chan, Toronto, UBC, Karolinska, Melbourne, Sydney, Queensland, Auckland, LSHTM, UCL, Copenhagen, Maastricht. AUB includes Social Entrepreneurship as an elective. Columbia Mailman’s Fast-Pitch, NUS TechLaunch, and UCT link entrepreneurship to applied projects. | Entrepreneurship remains optional in most programs and is rarely mandatory for all MPH students. |
| Educational Strategies / Pedagogy | Becker et al. (2019) use case methods and venture formation. Hernández et al. (2014) use seminars. Hyde et al. (2024) highlight sustainability challenges. | Columbia Mailman’s Fast-Pitch, ASU’s design-thinking curriculum, AUB’s studio formats, NUS TechLaunch’s incubator model, Karolinska’s commercialization focus, Harvard Chan’s case methods. Electives at Toronto, UBC, UCL, Copenhagen, Maastricht, LSHTM, Melbourne, Sydney, Queensland, Auckland. UCT links practice projects to innovation hubs. | Experiential and mentor-supported learning dominates, but sustainability is challenged by resource constraints. |
| Entrepreneurial Skills Addressed | Hyde et al. (2024) highlight innovation and mentorship. Hernández et al. (2014) identify legal, marketing, and finance domains. Becker et al. (2019) note gaps in team formation and management. Communication and stakeholder engagement emphasized by Becker et al. (2019). Evaluation and accountability included by Hernández et al. (2014) and Becker et al. (2019). | Harvard Chan/HBS on business modeling; Columbia Mailman on ideation and pitching; Johns Hopkins on low-resource innovation; Karolinska on IP and regulatory navigation; NUS TechLaunch on venture formation; ASU on design thinking; AUB on social venture design; UCT on LMIC systems. Electives at Toronto, UBC, UCL, Copenhagen, Maastricht, LSHTM, Melbourne, Sydney, Queensland, Auckland. | Skill overlap is strong, but competency frameworks and progression levels are rarely articulated. |
| Outcomes / Impact | Becker et al. (2019) align entrepreneurship with CEPH reforms. Hyde et al. (2024) note fragile sustainability. Hernández et al. (2014) suggest career diversification. | Columbia Mailman and ASU’s applied training; Central American Fellowship’s implementation; Thailand’s validated curriculum. Harvard Chan, Karolinska, NUS, and others use project-based assessments. Longitudinal tracking of outcomes is rare. | Impact measurement relies on proximal outcomes; sustainability depends on external support and institutional commitment. |
| Challenges and Facilitators | Hyde et al. (2024) cite faculty capacity and curricular priorities. Becker et al. (2019) advocate inter-professional training. Hernández et al. (2014) propose seed funding and metrics. | Harvard Chan and UCL use innovation hubs. Karolinska and NUS link faculties for commercialization. Columbia Mailman connects students with industry mentors. AUB and UCT anchor offerings in social-enterprise networks. Elective-only programs (Toronto, UBC, Australia/NZ) limit reach. | Enablers include mentorship, networks, and experiential formats; scaling and LMIC adaptation remain under-documented. |
